# Harnessing autophagy to overcome mitogen‐activated protein kinase kinase inhibitor‐induced resistance in metastatic melanoma

**DOI:** 10.1111/bjd.17333

**Published:** 2018-11-25

**Authors:** S. Verykiou, M. Alexander, N. Edwards, R. Plummer, B. Chaudhry, P.E. Lovat, D.S. Hill

**Affiliations:** ^1^ Institute of Cellular Medicine Newcastle University The Medical School Framlington Place Newcastle upon Tyne NE2 4HH U.K; ^2^ Northern Institute for Cancer Research Newcastle University The Medical School Framlington Place Newcastle upon Tyne NE2 4HH U.K; ^3^ Institute of Genetic Medicine Newcastle University Central Parkway Newcastle upon Tyne NE1 3BZ U.K

## Abstract

**Background:**

Patients with malignant melanoma often relapse after treatment with BRAF and/or mitogen‐activated protein kinase kinase (MEK) inhibitors (MEKi) owing to development of drug resistance.

**Objectives:**

To establish the temporal pattern of CD271 regulation during development of resistance by melanoma to trametinib, and determine the association between development of resistance to trametinib and induction of prosurvival autophagy.

**Methods:**

Immunohistochemistry for CD271 and p62 was performed on human naevi and primary malignant melanoma tumours. Western blotting was used to analyse expression of CD271, p62 and LC3 in melanoma subpopulations. Flow cytometry and immunofluorescence microscopy was used to evaluate trametinib‐induced cell death and CD271 expression. MTS viability assays and zebrafish xenografts were used to evaluate the effect of CD271 and autophagy modulation on trametinib‐resistant melanoma cell survival and invasion, respectively.

**Results:**

CD271 and autophagic signalling are increased in stage III primary melanomas vs. benign naevi. *In vitro* studies demonstrate MEKi of *BRAF*‐mutant melanoma induced cytotoxic autophagy, followed by the emergence of CD271‐expressing subpopulations. Trametinib‐induced CD271 reduced autophagic flux, leading to activation of prosurvival autophagy and development of MEKi resistance. Treatment of CD271‐expressing melanoma subpopulations with RNA interference and small‐molecule inhibitors to CD271 reduced the development of MEKi resistance, while clinically applicable autophagy modulatory agents – including Δ9‐tetrahydrocannabinol and Vps34 – reduced survival of MEKi‐resistant melanoma cells. Combined MEK/autophagy inhibition also reduced the invasive and metastatic potential of MEKi‐resistant cells in an *in vivo* zebrafish xenograft.

**Conclusions:**

These results highlight a novel mechanism of MEKi‐induced drug resistance and suggest that targeting autophagy may be a translatable approach to resensitize drug‐resistant melanoma cells to the cytotoxic effects of MEKi.

## Abstract 2

**What's already known about this topic?**

- The targeted mitogen‐activated protein kinase kinase (MEK) inhibitor (MEKi) trametinib is used for the treatment of patients with *BRAF*‐mutant metastatic melanoma in combination with BRAF inhibitors, but many tumours rapidly develop resistance to these drugs.
- Resistance of melanoma to trametinib has been associated with the induction of prosurvival autophagy, but it is unclear how stem‐cell markers, such as CD271 contribute to the transitionary phase of drug resistance.

**What does this study add?**

- Transient induction of the low‐affinity neurotrophin receptor CD271 is a critical regulator of the adaptive drug‐‐response phase of melanoma cells to trametinib.
- Genetic or chemical inhibition of CD271 prevents emergence of trametinib‐induced drug‐resistant melanoma subpopulations.
- Progression to trametinib resistance by melanoma subpopulations results in loss of CD271 expression and subsequent resistance to CD271 inhibitors, but not to the cytotoxic effects of autophagy modulation.

**What is the translational message?**

- Autophagy modulation either through inhibition of vacuolar protein sorting 34, or activation of cytotoxic autophagy with tetrahydrocannabinol effectively resensitizes drug‐resistant melanoma cells to trametinib *in vitro* and *in vivo*.
- Combined autophagy modulation and MEK inhibition offers a novel personalized therapeutic strategy to overcome MEKi‐induced drug resistance for patients with *BRAF*‐mutant metastatic melanoma.

Plain language summary available online

Respond to this article

Cutaneous melanoma remains the most deadly form of skin cancer with a rapidly increasing global incidence.^1^ Oncogenic *BRAF* mutations, such as the common V600E substitution present in 30–50% of melanomas.^2^, ^3^ activate the downstream effector mitogen‐activated protein kinase kinase (MEK) to drive melanoma tumour growth. Despite improvements in clinical outcome since the introduction of selective BRAF and MEK inhibitors (BRAFi/MEKi) and immune checkpoint inhibitors, development of resistance to these drugs remains a major problem, with little improvement in overall patient survival.^4^, ^5^, ^6^ Proposed mechanisms of resistance to BRAFi/MEKi treatment include selection of melanoma subpopulations with inherent genetic/epigenetic changes,^7^, ^8^ or the induction of generic stress‐induced response mechanisms that raise the survival threshold of the cell.^9^, ^10^, ^11^ This manuscript describes how two complementary mechanisms – CD271 signalling and autophagy – interact to regulate resistance of metastatic melanoma to the MEKi trametinib, as well as how they may be targeted to overcome drug resistance as an improved therapeutic approach for patients with melanoma.

CD271 (low‐affinity nerve growth factor receptor, nerve growth factor receptor or p75 neurotrophin receptor) is a member of the tumour necrosis factor superfamily that is critical in determining cell survival or death decisions.^12^ Previous studies indicate that CD271 expression within primary melanomas correlates with a more aggressive tumour phenotype and reduced patient survival,^13^ while isolated CD271‐expressing cells from primary melanomas initiate tumour growth in immunocompromised NRG mice at a higher rate than CD271^–^ cells.^14^ Conversely, other studies suggest that, rather than being a marker of distinctive melanoma‐initiating subpopulations, CD271 expression is induced during acquired resistance to BRAF inhibition, DNA‐damaging drugs or ethanol.^15^, ^16^, ^17^, ^18^, ^19^ Collectively, these studies indicate that CD271 is a biomarker of tumour progression and is consistent with the concept that CD271 signalling constitutes a stress‐tolerance mechanism within tumour cells.

Autophagy is the principal intracellular signalling mechanism, responsible for the degradation and recycling of damaged and/or excess proteins and organelles, and serves as a critical regulator of the survival/death response of melanoma cells.^20^, ^21^ Previous studies have shown that BRAF inhibition upregulates autophagy in patients with *BRAF*‐mutant melanoma, whereas inhibition of autophagic flux with the lysosomal inhibitor chloroquine (CQ) overcomes BRAF inhibitor‐induced resistance in several cancers, including brain, colorectal and melanoma,^22^, ^23^, ^24^ suggesting that autophagy inhibition may be beneficial for the control of drug‐resistant tumours in general. Conversely, as autophagy also has a tumour‐suppressor function, activation of autophagy, for example by cannabinoids such as Δ9‐tetrahydrocannabinol (THC) or cannabidiol, has been shown to induce cytotoxicity of melanoma,^25^, ^26^ which may potentially overcome the resistance of melanoma to BRAF/MEK inhibition.

In the present study we demonstrate that the induction of CD271 and modulation of autophagy are part of a temporary innate response by melanoma cells to drug‐induced stress that characterizes a stress‐tolerant state preceding development of drug resistance and tumour progression. Single‐agent trametinib was used as a time‐efficient model for development of drug resistance, which also occurs in response to BRAF inhibition or combined BRAFi + MEKi treatment at longer time points.^17^ We show that inhibiting either CD271 or autophagy using clinically relevant small‐molecule inhibitors effectively targets the stress‐tolerant state to induce death of MEKi‐resistant melanoma subpopulations. Moreover, using an *in vivo* zebrafish xenograft of human melanoma we show reduced metastatic potential of MEKi‐resistant melanoma cells in response to combined treatment with trametinib and a novel Vps34 inhibitor PIK‐III,^27^ while, importantly, having no adverse effect on the development and survival of the zebrafish. This work underpins the clinical relevance of CD271 and autophagy inhibition as a strategy to overcome the acquired resistance of *BRAF*‐mutant melanoma to mitogen‐activated protein kinase pathway inhibitors.

## Materials and methods

### Use of patient‐derived tissue

Written informed consent and full ethical permission for the use of patient tissue, including naevi (benign, noninvasive), primary cutaneous melanomas and normal melanocytes, was obtained from the Newcastle and Tyneside 1 research ethics committee in accordance with the Declaration of Helsinki Principles (reference: NRES 08/H0906/95 + 5).

### Immunohistochemistry

A retrospective cohort of formalin‐fixed paraffin‐embedded (FFPE) benign naevi or American Joint Committee on Cancer (AJCC) stage I, II or III primary melanomas at diagnosis (see Appendix S1 for classification details; Supporting Information)^28^ were obtained from the histopathology departments at the Royal Victoria Infirmary (Newcastle upon Tyne) and the University Hospital of North Durham (Durham). Semi‐quantitative immunohistochemical analysis of CD271 and p62 was performed according to a preoptimized methodology, as described in Appendix S1 (see Supporting Information).

### *In vitro* culture of human cell lines

Cutaneous human metastatic melanoma cell lines WM35 (a generous gift from Professor Meenhard Herlyn, The Wistar Institute, Philadelphia, PA, U.S.A.), A375 and SKmel28 (American Type Culture Collection, Manassas, VA, U.S.A) were grown and maintained as described in Appendix S1 (see Supporting Information). The authenticity of all cell lines was verified by melanA staining and confirmation of NRAS/BRAF mutational status using custom TaqMan SNP genotyping assays (Applera, Europe BV, Cheshire, U.K.) for the presence of the most frequent mutations observed in melanomas – *NRAS* ^Q61L^, *NRAS* ^Q61A^, *BRAF* ^V600E^ or *BRAf* ^V600D^ – as previously described,^29^ and cultures were maintained for up to a maximum of 50 passages. For long‐term trametinib treatment of melanoma cell lines, each cell line was cultured in complete culture media containing 16 nmol L^−1^ trametinib, which was changed every 3 days. When trametinib‐resistant melanoma subpopulations emerged they were passaged by trypsinization as normal and maintained in complete culture media containing fresh 16 nmol L^−1^ trametinib.

### Zebrafish xenograft assay

Transparent transgenic *fli1‐GFP* Casper zebrafish were housed under standard conditions on a constant 14 h on/10 h off light cycle at 28·5°C. All animals were maintained according to the ARRIVE guidelines under U.K. Home Office project licence 604548 held by B.C, which adhere to the requirements of the Animals (Scientific Procedures) Act 1986 of the U.K. Government and conformed to Directive 2010/63/EU of the European Parliament. For full details of xenograft assay see Appendix S1 (see Supporting Information).

Please see Appendix S1 (see Supporting Information) for additional details.

## Results

### CD271 and autophagy in primary and metastatic melanoma

**Figure 1:**
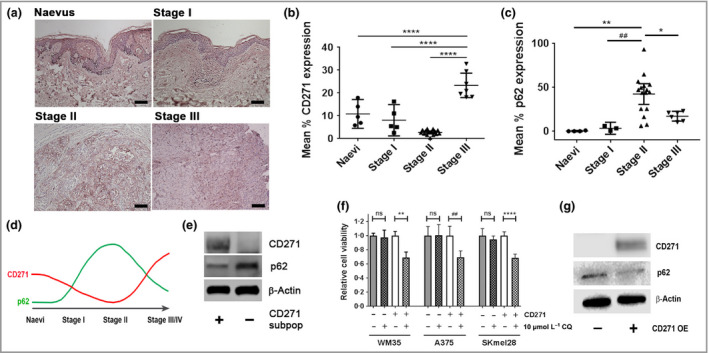
Expression and correlation of CD271 and the autophagy marker p62 in primary melanomas and metastatic melanoma cell lines. (a) Representative photomicrographs of immunohistochemical CD271 staining (pink) in a human benign naevus and American Joint Committee on Cancer (AJCC) stage I–III primary melanomas of increasing Breslow depth and spread (scale bars = 100 μm). Semi‐quantitative scoring of (b) CD271 and (c) p62 expression in naevi (*n *=* *5) and AJCC stage I (*n *=* *5), stage II (*n *=* *15) and stage III (*n *=* *7) primary melanomas. Each point is the mean CD271 or p62 expression of 10 images taken of each naevus or primary melanoma ± 95% confidence interval [CI; one‐way anova with Tukey's post‐hoc correction (*P =* *0·027, **P =* *0·001, ^##^ *P *=* *0·007, ****P <* *0·001)]. (d) Schematic of observed pattern of CD271 expression (red line) during melanoma progression vs. previously reported biphasic expression of p62 (green line). (e) Representative Western blot analysis of CD271 (75 KDa), p62 (65 KDa) and β‐actin (42 KDa) expression in subpopulations of the WM35 metastatic melanoma cell line isolated using CD271 antibody‐tagged microbeads and magnetic separation columns. (f) Relative viability, as measured by 3‐(4,5,dimethythiazol‐2‐y)‐5‐(3‐carboxymethoxy‐phenyl)‐2‐(4‐sulfophenyl)‐2H‐tetrazolium, inner salt (MTS) metabolism, of isolated CD271^+^ and CD271^–^ subpopulations from WM35, A375 and SKmel28 melanoma cell lines following 24‐h treatment in the presence or absence of 10 μmol L^−1^ chloroquine (CQ) [*n *=* *18 replicates from three independent experiments; mean ± 95% CI, one‐way anova with Tukey's post‐hoc correction (**P =* *0·003, ^##^ *P *=* *0·001, ****P <* *0·001)]. ns, nonsignficant. (g) Representative Western blot analysis of CD271 (75 KDa), p62 (65 KDa) and β‐actin (42 KDa) expression in WM35 cells before and after lentiviral‐mediated overexpression of CD271 (CD271 OE).

The prognostic potential of CD271 and p62 as biomarkers of disease progression was determined by their immunohistochemical expression in a cohort of FFPE primary naevi and melanomas of differing AJCC disease stage (Fig. 1a–c).^28^, ^30^ CD271 expression was significantly greater in stage III primary melanomas compared with naevi, stage I or stage II melanoma (all *P *<* *0·001; Fig. 1b). Lowest CD271 expression was observed in stage II melanomas, which was associated with increased p62 expression (*P *=* *0·001; Fig. 1c).^30^ These data suggest that AJCC stage‐dependent CD271 expression correlates with the level of p62 expression in melanomas (Fig. 1d). Western blotting demonstrated variable CD271 expression in a panel of metastatic melanoma cell lines, with greatest basal expression in *BRAF* ^V600I^‐mutant WM35 melanoma cells (Fig. S1; see Supporting Information), while expression in *BRAF* ^V600E^‐mutant A375 and SKmel28 melanoma cells was comparable to primary human melanocytes. Western blot analysis also demonstrated CD271^+^ subpopulations isolated from the WM35 cell line using CD271 antibody‐tagged microbeads and magnetic separation columns had reduced p62 expression vs. CD271^–^ cells (Fig. 1e), suggesting CD271‐expressing subpopulations have increased basal autophagy.

To determine the contribution of increased autophagy to the survival of CD271‐expressing subpopulations, CD271^+^ cells isolated from A375, WM35 and SKmel28 cell lines were treated for 24 h with 10 μmol L^−1^ CQ, which inhibits autophagy by blocking lysosome/autophagosome fusion. Analysis of 3‐(4,5,dimethythiazol‐2‐y)‐5‐(3‐carboxymethoxy‐phenyl)‐2‐(4‐sulfophenyl)‐2H‐tetrazolium, inner salt (MTS) metabolism demonstrated that CQ significantly inhibited the viability of CD271^+^ cells (Fig. 1f), indicating CD271^+^ subpopulations require autophagy for survival.

To determine the effect of increased CD271 expression on p62, CD271 was exogenously overexpressed in WM35 cells. Western blot analysis demonstrated reduced p62 expression in WM35 cells overexpressing CD271 vs. WM35 wild‐type cells (Fig. 1g). The reduction in p62 is likely attributable to CD271 signalling rather than generic stress resulting from the transfection process as expression of several endoplasmic reticulum stress‐response genes [*ATF4*,* XBP1*,* ATF6*,* TRB3* and *DDIT3* (*CHOP*)] were not significantly altered (Fig. S1; see Supporting Information).

### CD271 expression and autophagic activity in response to trametinib

**Figure 2:**
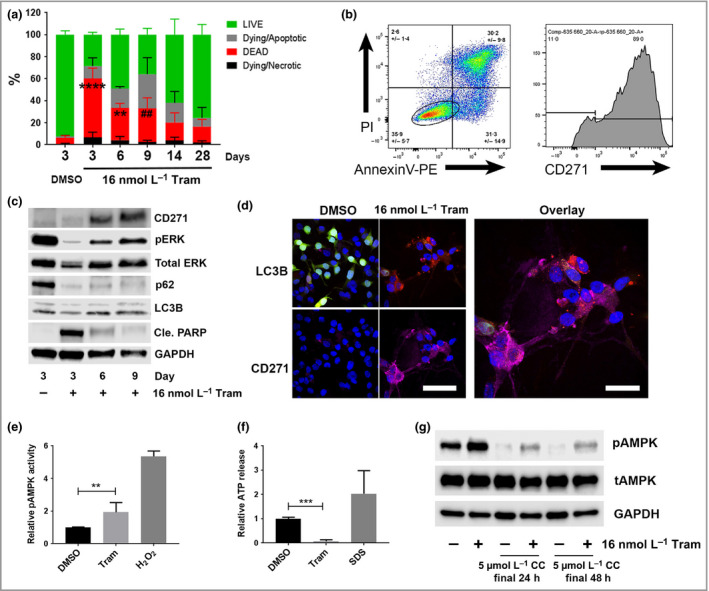
Trametinib induces CD271 expression and autophagic activity in melanoma cells *in vitro*. (a) Flow cytometry of AnnexinV–phycoerythrin (PE)/propidium iodide (PI)‐stained A375 melanoma cells for percentage live, dead, dying/apoptotic or dying/necrotic cell fractions in the presence or absence of treatment with 16 nmol L^−1^ trametinib (Tram) for 3, 6, 9, 14 or 28 days compared vs. dimethyl sulfoxide (DMSO) treatment (percentages did not significantly change between time points following DMSO treatment, which is shown at day 3 only for clarity) [*n *=* *3 independent experiments; mean ± 95% confidence interval (CI), two‐way anova with Tukey's post‐hoc correction for comparison of dead cells (^##^ *P *=* *0·007, **P =* *0·001, ****P <* *0·001)]. (b) Representative flow cytometry plots of A375 cells stained with AnnexinV–PE/PI/anti‐CD271 following treatment for 9 days with 16 nmol L^−1^ trametinib (mean ± SD of three independent experiments). An oval gate over ‘live cells’ was used to quantify CD271 expression, shown in the right‐hand panel. (c) Representative Western blot analysis for CD271 (75 KDa), phospho‐extracellular signal‐regulated kinase [ERK (pERK); 42/44 KDa], total‐ERK (42/44 KDa), p62 (65 KDa), LC3B (LC3‐I upper band, 14 KDa; LC3‐II lower band, 16 KDa), cleaved poly (ADP‐ribose) polymerase (Cle. PARP; 85 KDa) and glyceraldehyde 3‐phosphate dehydrogenase (GAPDH; 37 KDa) expression by A375 cells following treatment with DMSO or 16 nmol L^−1^ trametinib for 3, 6 or 9 days. (d) Representative fluorescent photomicrographs of LC3–red fluorescent protein (RFP)–green fluorescent protein (GFP) expressing WM266‐4 melanoma cells stained with anti‐CD271 (purple) following treatment for 9 days with DMSO or 16 nmol L^−1^ trametinib. LC3–RFP–GFP appears in cells as diffuse yellow/green fluorescence in the absence of autophagy but marks autolysosomes with red fluorescent puncta during active autophagy (scale bars = 50 μm). (e, f) Relative phospho‐adenosine monophosphate‐activated protein kinase (AMPK) activity or adenosine triphosphate (ATP) release by A375 cells following treatment with 16 nmol L^−1^ trametinib (Tram) or DMSO for 9 days, or 10 min in the presence of either 1 mmol L^−1^ H_2_O_2_ or 10% sodium dodecyl sulfate (SDS) as positive controls [*n *=* *3 independent experiments; mean ± 95% CI, one‐way anova with Tukey's post‐hoc correction (**P =* *0·002, ***P <* *0·001)]. (g) Representative Western blot analysis for phospho‐AMPK (pAMPK; 62 KDa), total AMPK (tAMPK; 62 KDa) and GAPDH (37 KDa) expression by A375 cells following treatment with 16 nmol L^−1^ trametinib for 9 days, alone or in the presence of 5 μmol L^−1^ Compound C (CC) for the final 24 or 48 h.

Dose‐escalation studies demonstrated that a clinically achievable concentration of 16 nmol L^−1^ trametinib induced 50–60% cell death of *BRAF* ^V600^‐mutant melanoma cells after 3 days. Flow cytometry analysis of annexin V/propidium iodide (PI)‐stained A375 cells treated with 16 nmol L^−1^ trametinib for 28 days demonstrated induction of apoptosis up to 14 days post‐treatment (Fig. 2a). Gating of live A375 cells co‐stained with annexinV/PI and anti‐CD271 antibody revealed the expression of CD271 on live melanoma subpopulations following exposure to trametinib for 9 days (Fig. 2b). Trametinib also induced the time‐dependent expression of CD271 in A375, WM35 and SKmel28 cells, with a concurrent reduction in p62 and increase in LC3‐II expression after 9 days of treatment, indicating co‐activation of autophagy and CD271 signalling (Fig. 2c, Fig. S2; see Supporting Information). Treatment of WM266‐4 *BRAF* ^V600D^‐mutant melanoma cells, stably expressing the dual‐tagged LC3–red fluorescent protein–green fluorescent protein reporter construct,^31^, ^32^, ^33^ with trametinib for 9 days also revealed an increase in red puncta and the expression of CD271 in individual cells, also indicating co‐activation of autophagy and CD271 signalling (Fig. 2d, Fig. S3; see Supporting Information). Treatment of A375 and WM35 cells with trametinib for 9 days increased activity of adenosine monophosphate‐activated protein kinase (AMPK), a known inducer of autophagy (Fig. 2e, Fig. S3; see Supporting Information) and reduced adenosine triphosphate (ATP) release (Fig. 2f, Fig. S2; see Supporting Information), both of which indicate a switch to catabolic, prosurvival metabolism. Furthermore, Western blot analysis demonstrated that treatment of A375 cells with trametinib for 9 days increased phosphorylation of AMPK, which was reversed by treatment with dorsomorphin (Compound C, an ATP mimic that inhibits AMPK)^34^ for the final 24 or 48 h (Fig. 2g). Collectively, these results indicate that prolonged MEKi in *BRAF*‐mutant melanoma cells induces CD271 and AMPK phosphorylation, while also increasing autophagy and disrupting cellular energy production.

### Effect of CD271 inhibition on cell viability and autophagic activity

**Figure 3:**
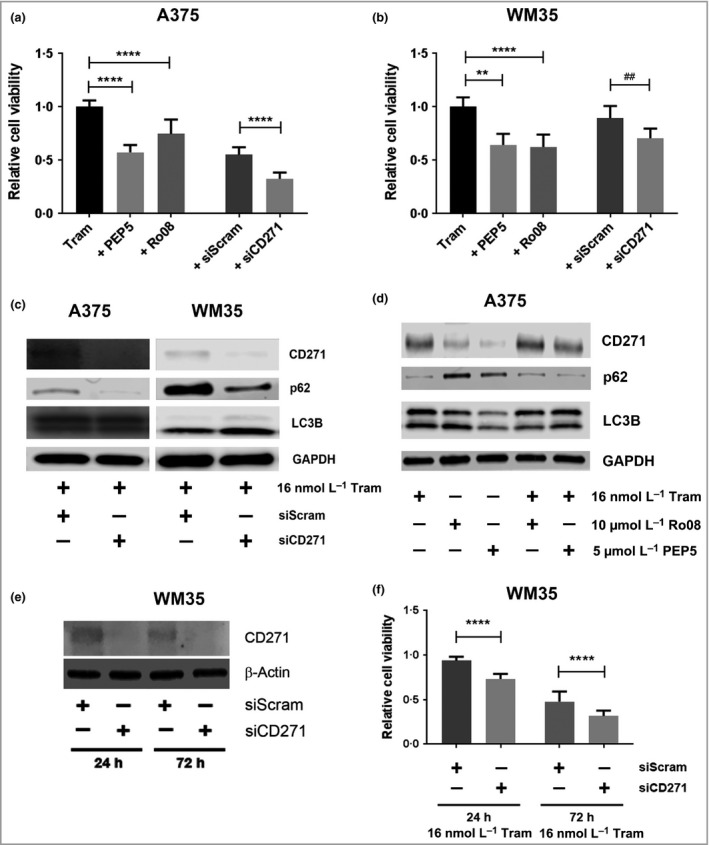
CD271 inhibition increases trametinib‐induced loss of melanoma cell viability *in vitro*. (a, b) Relative viability of A375 and WM35 melanoma cells following treatment with 16 nmol L^−1^ trametinib (Tram) for 9 days and then for a further 72 h in the presence or absence of 5 μmol L^−1^ TAT‐PEP5 (PEP5) or 10 mmol L^−1^ Ro08‐2750 (Ro08) CD271 inhibitors, or following transfection of scrambled nontarget (siScram) or CD271 (siCD271) small interfering RNA (siRNA) [*n *=* *18 replicates from three independent experiments; mean ± 95% confidence interval (CI), one‐way anova with Tukey's post‐hoc correction for CD271 inhibitors (**P =* *0·001, ****P <* *0·001), or unpaired *t*‐test for siRNA (^##^ *P *=* *0·009, ****P <* *0·001)]. (c) Representative Western blot analysis for CD271 (75 KDa), p62 (65 KDa), LC3B (14/16 KDa) and glyceraldehyde 3‐phosphate dehydrogenase (GAPDH; 37 KDa) expression by A375 or WM35 cells following pretreatment with 16 nmol L^−1^ trametinib for 9 days, transfection with siScram or siCD271, and continued treatment with 16 nmol L^−1^ trametinib (Tram) for a further 72 h. (d) Representative Western blot analysis for CD271 (75 KDa), p62 (65 KDa), LC3B (14/16 KDa) and GAPDH (37 KDa) expression by A375 cells following treatment with 16 nmol L^−1^ trametinib (Tram) for 9 days and then for a further 72 h in the presence or absence of 5 mmol L^−1^ PEP5 or 10 mmol L^−1^ Ro08. (e) Representative Western blot analysis for CD271 (75 KDa) and β‐actin (42 KDa) expression by WM35 cells following transfection with siScram or siCD271 for 24 or 72 h. (f) Relative viability, as measured by 3‐(4,5,dimethythiazol‐2‐y)‐5‐(3‐carboxymethoxy‐phenyl)‐2‐(4‐sulfophenyl)‐2H‐tetrazolium, inner salt (MTS) metabolism, of WM35 cells following transfection with siScram or siCD271 for 24 or 72 h in the presence of 16 nmol L^−1^ trametinib (Tram) relative to dimethyl sulfoxide‐treated cells at either time point (*n *=* *9 replicates from three independent experiments; mean ± 95% CI, one‐way anova with Tukey's post‐hoc correction ****P <* *0·001).

To determine whether trametinib‐induced CD271 functions as a prosurvival signalling mechanism contributing to drug tolerance, A375 and WM35 cells, pretreated for 9 days with 16 nmol L^−1^ trametinib, were further treated for 72 h with trametinib in combination with either the CD271 inhibitors TAT‐PEP5 (PEP5) or Ro08‐2750 (Ro08), or, alternatively, with small interfering RNA (siRNA) targeting CD271 (siCD271). Subsequent analysis of MTS metabolism demonstrated that chemical inhibition or siCD271 reduced the viability of trametinib‐treated A375 and WM35 cells vs. trametinib treatment alone or following transfection with a scrambled siRNA, respectively (Fig. 3a, b). Western blot analysis demonstrated that siCD271 of A375 or WM35 cells following treatment with trametinib for 9 days reduced p62 and increased LC3‐II expression vs. a scrambled siRNA, indicating increased autophagy in response to loss of CD271 (Fig. 3c, Fig. S4; see Supporting Information). Conversely, CD271 inhibitors did not appear to alter p62 or LC3‐II expression of A375 cells following treatment with trametinib for 9 days (Fig. 3d), suggesting PEP5 and Ro08 may reduce cell viability through inhibition of multiple mechanisms. In contrast to previous studies,^17^ overexpression of CD271 in A375 and WM35 cells did not reduce trametinib‐mediated inhibition of cell viability (Fig. 1f, Fig. S4; see Supporting Information).

To determine the effect of basal CD271 expression on response to trametinib, WM35 cells (with highest basal CD271 expression) were transfected with siCD271 prior to trametinib treatment. Western blot analysis confirmed knockdown of CD271 expression following siCD271 in WM35 cells for 24 or 72 h compared with cells transfected with a scrambled siRNA (Fig. 3e). Subsequent analysis of MTS metabolism demonstrated siCD271 for 24 h prior to treatment with trametinib for 24 or 72 h increased trametinib‐mediated inhibition of cell viability vs. cells transfected with a scrambled siRNA (Fig. 3f). Collectively, these data suggest that in the context of MEKi, CD271 contributes to development of drug resistance, potentially mediated through reduced cytotoxic autophagy.

### Harnessing autophagy modulation for the treatment of mitogen‐activated protein kinase kinase inhibitor‐resistant melanoma cells

**Figure 4:**
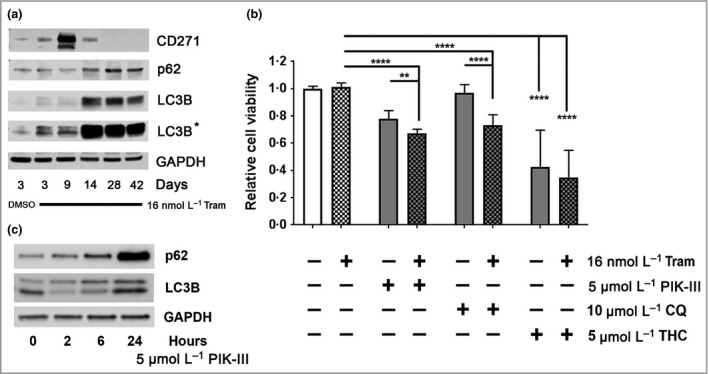
Inhibition of autophagy reduces the viability of trametinib‐resistant melanoma cells. (a) Representative Western blot analysis for CD271 (75 KDa), p62 (65 KDa), LC3B (14/16 KDa) and glyceraldehyde 3‐phosphate dehydrogenase (GAPDH; 37 KDa) expression by A375 cells following treatment with dimethyl sulfoxide (DMSO) or 16 nmol L^−1^ trametinib (Tram) for 3, 9, 28 or 42 days. (b) Relative viability, as measured by 3‐(4,5,dimethythiazol‐2‐y)‐5‐(3‐carboxymethoxy‐phenyl)‐2‐(4‐sulfophenyl)‐2H‐tetrazolium, inner salt (MTS) metabolism, of A375 cells following treatment with 16 nmol L^−1^ trametinib (Tram) for 42 days then for a further 48 h in the presence or absence of 16 nmol L^−1^ trametinib alone or in combination with 5 mmol L^−1^ PIK‐III, 10 mmol L^−1^ chloroquine (CQ) or 5 mmol L^−1^ Δ9‐tetrahydrocannabinol (THC) [*n *=* *18 replicates from three independent experiments; mean ± 95% confidence interval, one‐way anova with Tukey's post‐hoc correction (**P =* *0·005, ****P <* *0·001)]. (c) Representative Western blot analysis for p62 (65 KDa), LC3B (14/16 KDa) and GAPDH (37 KDa) expression by A375 cells following treatment with 5 mmol L^−1^ PIK‐III (Vps34 inhibitor) for 2, 6 or 24 h.

Western blot analysis of A375 cells treated with 16 nmol L^−1^ trametinib for 42 days demonstrated that MEKi‐induced CD271 expression peaked at approximately 9 days and returned to baseline by 28 days (Fig. 4a). Peak CD271 expression was subsequently followed by increased p62 expression and accumulation of total LC3 with a reduced LC3‐II/LC3‐I ratio between 9 and 14 days (Fig. 4a, Fig. S5c; see Supporting Information), indicating reduced autophagy as previously described,^35^ which coincided with a return to full proliferative and invasive potential, confirming development of drug resistance. Previous studies suggest autophagy inhibition represents a viable therapeutic approach to overcome resistance of *BRAF* ^V600E^‐mutant brain tumour and melanoma cells to BRAF inhibition.^22^, ^24^ To determine the role of autophagy within MEKi‐resistant melanoma cells, A375 cells continuously treated with 16 nmol L^−1^ trametinib for 42 days were further treated for 48 h with trametinib alone or in combination with 5 μmol L^−1^ PIK‐III, 10 μmol L^−1^ CQ or 5 μmol L^−1^ THC. Subsequent analysis of MTS metabolism showed that inhibition of autophagy with PIK‐III or CQ, or, alternatively, activation of autophagy with THC, in combination with trametinib, reduced the viability of MEKi‐resistant melanoma cells (Fig. 4b). Western blot analysis of A375 cells treated with 5 μmol L^−1^ PIK‐III for 2, 6 or 24 h demonstrated a time‐dependent increase in p62 and reduction in the LC3‐II/LC3‐I ratio, indicating inhibition of autophagy by PIK‐III (Fig. 4c, Fig. S5d; see Supporting Information). Furthermore, combined treatment of MEKi‐resistant A375 cells with trametinib and CQ for 7 days reduced migration/invasion into collagen compared with single‐agent treatment (Fig. S5; see Supporting Information).^36^ Collectively, these results indicate that autophagy modulation can contribute to resistance of *BRAF*‐mutant melanoma cells to MEK inhibition.

### *In vivo* efficacy of autophagy inhibition in a zebrafish xenograft of human melanoma

**Figure 5:**
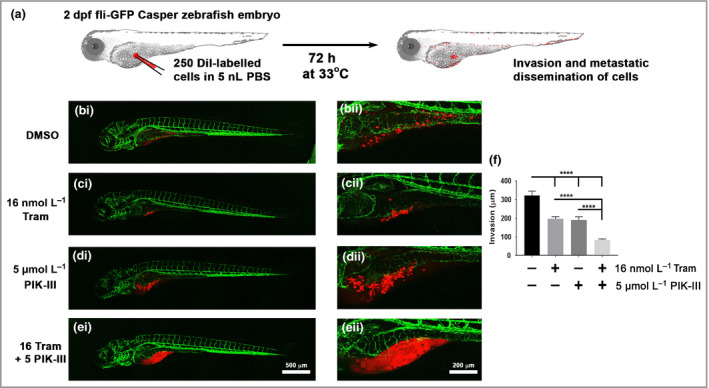
Inhibition of Vps34 reduces invasion and increases death of trametinib‐resistant melanoma cells in an *in vivo* zebrafish xenograft of metastatic melanoma. (a) Schematic of zebrafish xenograft assay showing injection of approximately 250 DiI‐labelled melanoma cells into the yolk sac of 2‐day postfertilization (dpf) zebrafish embryos prior to incubation at 33°C in atmospheric air for 72 h and subsequent analysis of melanoma invasion. (b–e) Representative confocal images of zebrafish embryos following injection with DiI‐labelled trametinib‐resistant A375 cell and treatment for 72 h with (bi, ii) dimethyl sulfoxide (DMSO), (ci, ii) 16 nmol L^−1^ trametinib (Tram), (di, ii) 5 μmol L^−1^ PIK‐III or (ei, ii) 16 nmol L^−1^ trametinib (Tram) + 5 μmol L^−1^ PIK‐III (16 Tram + 5 PIK‐III) by addition of drugs to E3 media. (f) Invasive distance of trametinib (Tram)‐resistant A375 cells from injection site [*n *=* *10 fish for each condition from five independent experiments; mean ± 95% confidence interval, Kruskal–Wallis test with Tukey's post‐hoc correction (****P <* *0·001)]. GFP, green fluorescent protein; PBS, phosphate‐buffered saline.

To evaluate whether combined autophagy and MEK inhibition reduces the invasive potential of MEKi‐resistant melanoma cells *in vivo*, we developed an embryonic zebrafish xenograft model of human metastatic melanoma in which DiI‐labelled melanoma cells were injected into the yolk sac of 2‐day postfertilization *fli1‐GFP casper* zebrafish embryos (Fig. 5a).^37^, ^38^, ^39^ Zebrafish injected with trametinib‐resistant A375 cells were treated with dimethyl sulfoxide, 16 nmol L^−1^ trametinib, 5 μmol L^−1^ PIK‐III or both drugs in combination for 3 days by addition of drugs to E3 aquarium water (Fig. 5b–e). Analysis of cell movement from site of injection demonstrated that combination treatment with trametinib and PIK‐III significantly reduced invasion of trametinib‐resistant A375 cells *in vivo* (Fig. 5f) compared with DMSO or single‐agent treatment. Moreover, the combination treatment regime induced cell death, as evidenced by the release of DiI from melanoma cells into the surrounding tissue. Importantly, the combination treatment regime was well tolerated and all fish survived treatment with no obvious developmental abnormalities after 3 days. Collectively, these results demonstrate that specific autophagy inhibition overcomes the metastatic and survival potential of MEKi‐resistant melanoma cells *in vitro* and *in vivo*.

## Discussion

This study aimed to determine mechanisms mediating the resistance of *BRAF*‐mutant melanoma cells to MEK‐specific inhibition and define innovative means through which to overcome such resistance in a clinical setting. Our observations of reduced CD271 expression in AJCC stage II melanomas suggest that CD271 may have a tumour‐suppressor function in normal melanocytes that is lost in the early stages of melanoma progression. However, increased CD271 expression in AJCC stage III tumours indicates that CD271 may potentially give tumour cells a survival advantage during metastasis.

We show that treatment of *BRAF*‐mutant melanoma cell lines with trametinib induced cytotoxic autophagy and subsequent cell death. However, prolonged exposure to trametinib led to the emergence of drug‐resistant melanoma subpopulations characterized by a transient increase in CD271 expression, the inhibition of which resulted in increased trametinib‐induced cell death, indicating that CD271 functions as a prosurvival mechanism in response to MEK inhibition. Trametinib‐induced CD271 signalling has recently been shown to induce prosurvival DNA damage response pathways,^18^ and data from the present study additionally suggest that such signalling may promote survival by inhibiting cytotoxic autophagy as knock‐down of trametinib‐induced CD271 in WM35 melanoma cells resulted in increased autophagy and reduced cell viability. However, results also suggest that CD271 may have opposing effects on autophagy in unstressed cells, such as CD271^+^ cells from treatment‐naïve cell lines that appear to require autophagy for survival vs. CD271^+^ cells undergoing drug‐induced stress, where CD271 appears to reduce cytotoxic autophagy. The role of CD271 as a component of diverse and/or opposing signalling networks is supported by recent studies characterizing CD271 as a regulator of the ‘phenotype switch’,^40^ and seems increasing likely given the large number of CD271‐interacting partners, as well as the ability of CD271 to function as a homodimer,^41^ or to elicit signalling in the absence of ligand binding.^42^

**Figure 6:**
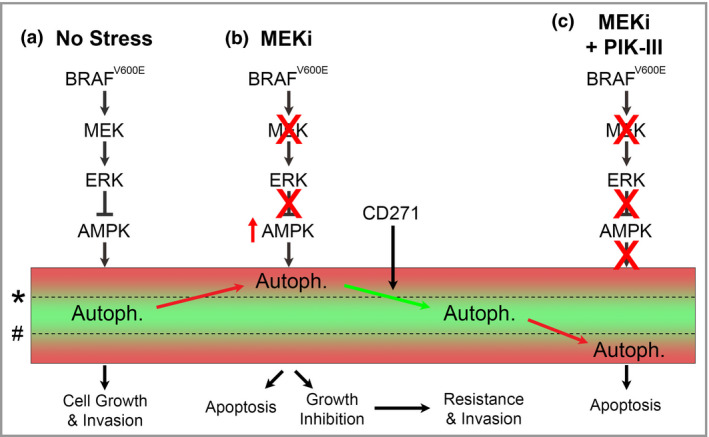
Hypothesized effect of mitogen‐activated protein kinase kinase (MEK) and autophagy inhibition on growth, survival and invasion of melanoma. (a) In normal growth conditions, activating *BRAF* mutations increase extracellular signal‐regulated kinase (ERK) signalling to inhibit adenosine monophosphate‐activated protein kinase (AMPK) and maintain autophagic activity (Autoph.) within survival limits. (b) MEK inhibitors (MEKi) de‐represses AMPK and allows autophagy to increase above the upper survival threshold (*) leading to activation of apoptosis or growth arrest. Subsequent activation of adaptive prosurvival signalling, such as CD271, contributes to drug resistance by lowering autophagic activity below the threshold for apoptosis, allowing reactivation of cell growth, proliferation and invasion. (c) Treatment of drug‐resistant melanoma with specific autophagy inhibitors, such as PIK‐III, reduces prosurvival autophagic activity below the lower survival threshold (#) leading to accumulation of cytotoxic by‐products and activation of apoptosis.

Data from the present study suggest a model whereby CD271 contributes to drug resistance by lowering autophagy below the threshold for induction of apoptosis allowing the cell to benefit from the prosurvival function of autophagy (Fig. 6). Therefore, similarly to BRAFi‐resistant brain tumour and melanoma cells,^22^, ^24^ it is likely that MEKi‐resistant melanoma subpopulations maintain a low autophagic activity to promote survival and metastasis, which can be subsequently targeted with CQ or by the novel, selective autophagy inhibitor PIK‐III to reduce invasion and viability of MEKi‐resistant melanoma cells both *in vitro* and *in vivo*. These results also support our previous findings showing that mutant *BRAF* suppresses autophagy,^43^ and provide a clear rationale for combining BRAFi/MEKi with clinically relevant inhibitors of autophagy such as those targeting the autophagy regulatory protein Vps34.

However, as autophagy is also a tumour‐suppressor mechanism in normal cells clinical inhibition of autophagy may sensitize patients to secondary tumour formation. Therefore, an alternative approach may be to exacerbate autophagy, for example with THC, which has been shown to activate cytotoxic autophagy through induction of endoplasmic reticulum stress, specifically in cancer cells, with minimal adverse effect on normal cell types.^25^, ^44^

This study highlights the great potential of harnessing the modulation of generic survival mechanisms, such as CD271 signalling and autophagy, as a means to overcome drug resistance and more effectively treat patients with currently incurable metastatic melanoma. However, for these approaches to be applied in the clinic, further *in vivo* validation, such as performed by recent studies demonstrating the potential of autophagy modulatory drugs in zebrafish,^45^ is required to confirm their antitumourigenic efficacy with minimal off‐target toxicity to normal cells. To this end, zebrafish are a useful and versatile model organism for the study of cancer that complement existing nonanimal models of melanoma, such as skin equivalents,^46^ by providing a fast, cost‐effective assay for studying *in vivo* melanoma metastasis and drug response.

Further preclinical studies of specific autophagy inhibitors, such as novel Vps34 inhibitors, or compounds and strategies that specifically mediate induction of cytotoxic autophagy in cancer cells, such as cannabinoids, will inform the design of early‐phase clinical trials and drive the efficacy of personalized targeted medicine that harnesses autophagy modulation of the benefit of patients with metastatic melanoma.

## Supporting information


**Appendix S1** Supplementary methods.

**Fig S1.** (a) Representative Western blot analysis of CD271 and β‐actin expression in primary human melanocytes (M1058F, M1054F, M880F) and human metastatic melanoma cell lines (WM35, A375 and SKmel28). (b) Densitometry quantification of CD271 expression and (c) quantification of CD271 mRNA transcript levels in primary human melanocytes and human metastatic melanoma cell lines (*n *=* *3 independent Western blot replicates). (d–h) Relative expression of Xbp1, ATF4, ATF6, TRB3 and CHOP mRNA transcript level in A375 melanoma cells flowing over expression of CD271 (CD271 OE).

**Fig S2.** (a) Densitometry quantification of CD271, p62 and LC3‐II expression by A375 cells following treatment with dimethyl sulfoxide or 16 nmol L^−1^ trametinib for 3, 6 or 9 days. (b, c) Representative Western blot analysis of CD271, p62 and LC3, and densitometry quantification of CD271, LC3‐II and p62 expression (*n *=* *3 independent Western blot replicates), by WM35 and SKmel28 cells.

**Fig S3.** (a–c) Mean fluorescence intensity of LC3–red fluorescent protein (RFP), LC3–green fluorescent protein (GFP) and CD271–A647 staining by WM266‐4 cells following treatment with 16 nmol L^−1^ trametinib for 9 days [*n *=* *10 images; mean ± 95% confidence interval (CI), Mann–Whitney test (*P =* *0·012, **P =* *0·002, ***P <* *0·001)]. (d) Relative phospho‐adenosine monophosphate‐activated protein kinase (AMPK) activity or (e) adenosine triphosphate (ATP) release by WM35 cells following treatment for 9 days in the presence or absence of 16 nmol L^−1^ trametinib, or 10 min in the presence of either 1 mmol L^−1^ H_2_O_2_ or 10% sodium dodecyl sulfate (SDS) as positive controls [*n *=* *3 independent experiments; mean ± 95% CI, one‐way anova with Tukey's post‐hoc correction, (*P =* *0·042, ****P <* *0·001)].

**Fig S4.** (a–d) Densitometry quantification of CD271, phospho‐adenosine monophosphate‐activated protein kinase (AMPK), p62 and LC3‐II expression by A375 and WM35 cells following treatment with 16 nmol L^−1^ trametinib for 9 days and then for a further 72 h following transfection with scrambled nontarget (siScram) or CD271 (siCD271) small interfering RNA (siRNA; *n *=* *3 independent Western blot replicates). (e) Densitometry quantification of CD271 and (f) quantification of CD271 mRNA transcript levels in WM35 cells after 24, 96 and 168 h after transfection with siScram or siCD271. (g, h) Relative viability, as measured by MTS metabolism, of WM35 and A375 wild‐type cells or cells overexpressing CD271, following treatment in the presence or absence of 16 nmol L^−1^ trametinib for 24, 48 or 72 h.

**Fig S5.** (a) Representative photomicrographs of A375 spheroids in type I collagen following treatment with 16 nmol L^−1^ trametinib for 42 days, and subsequently treated with dimethyl sulfoxide (DMSO), 16 nmol L^−1^ trametinib, 10 mmol L^−1^ chloroquine or the combination of 16 nmol L^−1^ trametinib and 10 mmol L^−1^ chloroquine [16 Tram + 10 chloroquine (CQ)] for 0, 2, 4 or 7 days; ×10 magnification, scale bar = 100 μm. (b) Invasion of A375 trametinib‐resistant spheroids relative to size at day 0 following the same treatment protocol as for (a) [*n *=* *24 cross‐sectional diameters at each time point from three independent experiments; mean ± SD, two‐way anova with Tukey's post‐hoc correction (**P =* *0·002, ***P <* *0·001)]. (c) Densitometric quantification of the ratio of LC3‐II/LC3‐I expression by A375 cells treated for 3 days with DMSO, or for 3, 9, 14, 28 and 42 days with 16 nmol L^−1^ trametinib (*n *=* *3 independent Western blot replicates). (d) Densitometric quantification of the ratio of LC3‐II/LC3‐I expression by A375 cells treated for 0, 2, 6 or 24 h with 5 mmol L^−1^ PIK‐III (*n *=* *3 independent Western blot replicates).
