# Supplementary material for: Harnessing autophagy to overcome mitogen‐activated protein kinase kinase inhibitor‐induced resistance in metastatic melanoma
Source: Br J Dermatol. 2018 Nov 25;180(2):346–56. doi: 10.1111/bjd.17333 (PMC7816093; doi:10.1111/bjd.17333)
Supplement: Supplementary file 1 — Appendix S1 Supplementary methods. [file BJD-180-346-s001.docx]

**Supplementary Methods**

**Immunohistochemistry** – Optimal antigen retrieval (10 mM Tris-HCl (pH9)) and primary antibody concentrations (1:50 in PBS/2%BSA) were pre-determined and then used to determine semi-quantitative expression of CD271 (Abcam Biochemicals, Cambridge, UK; ab31251) and p62 (Santa Cruz Biotenchnology, USA; SQSTM1 Antibody (D-3): sc-28359) in a cohort of 35 FFPE primary naevi/melanomas (5 benign naevi, 5 AJCC stage I, 16 AJCC stage II, 9 AJCC stage III). Following CD271 and p62 staining, representative images were captured using a Zeiss Axio Imager microscope (Carl Zeiss Microscopy New York, U.S.A), and digitally imaged for quantification using an automated slide scanner (Leica SCN400 digital slide scanner; Leica Biosystems, Milton Keynes, UK).

***In vitro* culture and drug treatment of human cell lines** – Melanoma cells were cultured in high glucose (4.5 g/L) Dulbecco’s modified Eagles Medium with L-glutamine (DMEM; Lonza, Vervies, Belgium) supplemented with 10% foetal calf serum (FCS; Sigma-Aldrich Ltd, Poole, UK) and 5% penicillin streptomycin (P/S, Lonza, Vervies, Belgium) (complete media). Primary melanocytes were isolated from foreskin tissue and cultured for a maximum of 5 passages in medium 254 (Life Technologies, Paisley, U.K), supplemented with 1% human melanocyte growth supplement (Life Technologies, Paisley, U.K). All cells were cultured at 37^o^C in a humidified incubator containing 5% CO_2_ in air.

Cell lines were used at approximately 70% confluency for all experimental procedures. Trametinib (Santa Cruz Biotechnology, Heidelberg, Germany) was dissolved in DMSO to 10 mM before further dilution to 10 μM in complete media as a working stock; chloroquine (Sigma-Aldrich Ltd, Poole, UK) was dissolved in water to 10 mM stock; 1 mM TAT-PEP5 (Merck Millipore, Massachusetts, USA), 5 mM Compound C (Stratech Scientific Limited, Suffolk, UK), 10 mM Ro08-2750 (Tocris Bioscience, Bristol, UK) and 10 mM PIK-III (Stratech Scientific Limited, Suffolk, UK) were prepared in DMSO. Drugs were added to complete media for *in vitro* cell treatment or to E3 media for *in vivo* zebrafish treatment at concentrations specified and an equal volume of DMSO vehicle was used to treat control cells or zebrafish for each experiment.

**Subpopulation isolation using magnetic microbeads** – Cell subpopulations were isolated on the basis of CD271 expression from WM35, A375 or SKmel28 melanoma cells using magnetic separation columns according to manufacturer’s instructions (Miltenyi Biotec Ltd; Auburn, USA). Briefly, 1×10^7^ cells in sterile PBS were filtered through 30 μm CellTrics pre-separation filters (Miltenyi Biotec), centrifuged at 310 g for 5 minutes, and re-suspended in 80 μl of fresh ice-cold wash buffer (2mM Ethylenediaminetetraacetic acid (EDTA), 0.5% BSA in PBS) containing 10 μl of human FcR blocking reagent (Miltenyi Biotec Ltd; Auburn, USA) and 10 μl of CD271-PE antibody (Miltenyi Biotec Ltd; Auburn, USA). Cell suspension was incubated for 10 minutes at 4°C, washed in 1 ml ice-cold wash buffer and centrifuged at 310 g for 5 minutes. Cells were re-suspended in 70 μl ice-cold buffer containing 10 μl of FcR blocking reagent and 20 μl of anti-PE microbeads (Miltenyi Biotec Ltd; Auburn, USA). Cell suspension was incubated for 15 minutes at 4°C, washed in 1 ml ice-cold wash buffer and centrifuged at 310 g for 5 minutes, prior to re-suspending cells in 1 ml wash buffer. The cell suspension was passed through an MS MiniMACS Separator column attached to a separation magnet (Miltenyi Biotec Ltd; Auburn, USA) to collect CD271 negative subpopulations prior to removing the column from magnet and adding 1 ml wash buffer to collect CD271 positive subpopulations, which were used for subsequent experiments and analysis.

**Western blotting** – Western blot analysis was performed according to a standardized protocol as previously described ^(^[^28^](#_ENREF_28)^)^. Briefly, total cell and supernatant cultures were solubilized at approximately 2x10^6^ cells per ml of ice-cold lysis buffer (0.1 M Tris-HCl pH 7.4, 25 mM NaF, 0.1 M NaCl, 2 mM EDTA (pH 8), 1 mM benzamidine, , 0.1% Triton-X100, 0.1 mM sodium orthovanadate and 1 x protease inhibitor cocktail (Promega, Southampton, UK). Protein lysates were sonicated (Soniprep150, MSE, UK) and quantified using a Bradford assay (Pierce Biotech, Rockford, USA) according to the manufacturers’ instructions. Protein lysate (10 µg) was loaded onto 4-20 % tris-glycine gels (Bio-Rad, Watford, UK) alongside a Benchmark pre-stained protein ladder (Invitrogen, Paisley, UK) and separated by electrophoresis prior to transfer onto a PVDF membrane using a Turbo-blotter (1.3A, 25V; Bio-Rad, Watford UK). Membranes were blocked in 5% non-fat milk dissolved in tris-buffered saline with 0.1% (v/v) Tween-20 (TBS/T) for 30 minutes at room temperature, then incubated over night at 4^o^C in primary antibody against either CD271 (1:5000 D8A8 #4201; Cell Signalling Technology, Danvers, Massachusetts, USA), LC3B (1:2000 #2775; Cell Signalling Technology), p62 (SQSTM1 D-3; 1:1000 sc-28359; Santa Cruz Biotechnology), cleaved caspase-3 (1:1000 #9664; Cell Signalling Technology), cleaved PARP (1:500 #9541; Cell Signalling Technology), ERK 1/2 (p44/42 MAPK; 1:5000 #9102; Cell Signalling Technology), phospho-ERK 1/2 (1:5000 #4370; Cell Signalling Technology), AMPKα (1:2000 #2532; Cell Signalling Technology), phospho-AMPKα (1:2000 #4188; Cell Signalling Technology), β−actin (1:40000 A5316; Sigma Aldrich) or GAPDH (1:10000 #2118; Cell Signalling Technology), diluted TBS/T containing 5% BSA.

**MTS cell viability assay** – Melanoma cells were seeded in complete culture media at a density of 5 or 2.5x10^3^ cells per well in a volume of 100 μl per well of a 96-flat well plate (Corning Incorporated, New York, U.S.A), with at least four replicates per experimental condition. Cells were treated in a volume of 100 µl for 24 or 48 hours at 37^o^C in a humidified incubator containing 5% CO_2_ in air. Cell viability was assessed by the addition of 20 µl of aqueous non-radioactive cell proliferation reagent 3-(4,5-dimethylthiazol-2-yl)-5-(3-carboxymethoxyphenyl)-2-(4-sulfophenyl)-2H tetrazolium (MTS; CellTitre96, Promega, Southampton, UK) to each well for 4 hours prior to detection of absorbance at 490 nm using a Spectra Max 250 plate reader (Molecular Devices, Wokingham, UK). Or alternatively, melanoma cells were seeded in complete culture media at a density of 5×10^4^ cells per well in a 12-flat well tissue culture plate (Corning Inc, Corning, NY, USA) and treated in a volume of 1 ml for 24 or 48 hours. Cell viability was assessed by addition of 100 µl of 5 mg/ml thiazolyl blue tetrazolium bromide (MTT; Sigma-Aldrich Ltd, Poole, UK) to each well for 4 hours, which was subsequently aspirated and replaced with 300 μL of isopropanol/hydrochloric acid (HCl) solution (5 ml HCL in 500 ml isopropanol) for 5 minutes prior to transferring 100 μL in triplicate to a 96-well plate and measuring absorbance at 570 nm using a Spectra Max 250 plate reader (Molecular Devices, Wokingham, UK).

**Flow cytometry for apoptosis and CD271 expression** – A375 cells were seeded at a density of 2×10^6^ well in T75 tissue culture flasks (Corning Incorporated, New York, USA) in a final volume of 10 ml of complete culture medium (DMEM, 10% FCS, 1% PS) alone or containing 16 nM trametinib for 3, 6, 9, 14, 28 or 42 days at 37^o^C in a humidified incubator containing 5% CO_2_ in air. CD271 expression was detected by binding of anti-CD271 antibodies (C40-1457; BD Pharmingen) and secondary antibody binding of goat anti-mouse Alexafluor633 (A-21052; Thermo Fisher Scientific, Paisley, UK), prior to detection of apoptosis using an Annexin V-Fluorescein (FITC)/propidium iodide (PI) detection kit (ab14085, Abcam, Cambridge, UK) according to the manufacturer’s protocol. Fluorophores were detected using a FACS-Canto II (8 Colour) flow cytometer (BD biosciences, San Jose, USA) and data was analysed using FlowJo analysis software for flow cytometry (FlowJo, Ashland, Oregon, USA).

**AMPK activity** – Melanoma cells were seeded at 2×10^6^ cells per 10 cm tissue culture plate for trametinib treatment or 2x10^4^ cells per plate for DMSO or hydrogen peroxide (H_2_O_2_)-treated controls in 7 ml of complete culture medium and incubated overnight at 37^o^C. Medium was replaced with fresh complete culture media containing DMSO (in duplicate for positive control) or 16 nM trametinib, and cells incubated for 9 days changing the medium and drug every 3 days. 10 mM hydrogen peroxide (Sigma Aldrich, St Louis, USA) was added to positive control plates and incubated for 10 mins at 37^o^C prior to removal of medium from all plates. Cells were subsequently washed in ice-cold PBS, lysed by addition of 500 μL of ice-cold Elisa Lysis buffer (Cell Signalling Technology, Danvers, Massachusetts, USA) and detached using cell scrapers. Cell lysates were incubated on ice for 30 min and protein concentration determined using a commercial Bradford assay. Protein lysate (10 µg) was added to a commercial sandwich enzyme-linked immunosorbent assay (ELISA) for detection of pAMPKα (Phospho-AMPKα (Thr172) Sandwich ELISA Kit #7959; Cell Signalling Technology, Danvers, USA) according to manufacturer’s instructions, and absorbance was measured at 450 nm using a Spectra Max 250 plate reader (Molecular Devices, Workingham, UK).

**ATP release** – Melanoma cells treated with DMSO or 16 nM trametinib for 8 days were seeded at a density of 5x10^3^ cells per well of an opaque-walled 96 flat well plate (Corning opaque 96-well plates, Sigma-Aldrich Ltd, Poole, UK) in 100 μl of complete medium containing DMSO or 16 nM trametinib with at least four replicates per experimental condition (duplicate DMSO wells seeded for positive control) and incubated overnight at 37^o^C. Medium was replaced with 100 µl of fresh media containing DMSO or 16 nM trametinib and incubated for 24 hours at 37^o^C. 10 µl of 10% sodium dodecyl sulphate (SDS; Sigma-Aldrich Ltd, Poole, UK) in de-ionized water were added to positive control wells for 2 minutes at room temperature prior to addition of 100μl of CellTiter-Glo reagent per well to all wells and measurement of luminescence using a GloMax® Explorer luminometer (Promega, Southampton, UK).

**Immunofluorescence** – Melanoma cells expressing an LC3-RFP-GFP reporter construct as previously described [28, 34, 35] were seeded onto glass cover slips (22x22 mm) at 5x10^4^ cells per well of a 6-well tissue culture plate (Corning Incorporated, New York, USA) prior to treatment with 16 nM trametinib for 9 days. Subsequently, cells were fixed with ice-cold paraformaldehyde (4%) in PBS for 15 minutes on ice, washed three times with PBS, permeabilised in 0.2% Triton X-100 (Sigma-Aldrich Ltd, Poole, UK) in phosphate-buffered saline with 0.1% (v/v) Tween-20 (PBS/T) for 10 minutes, and incubated with 2% goat serum (Sigma-Aldrich Ltd, Poole, UK) in PBS/T for 30 minutes at room temperature. CD271 was detected by incubating in anti-human CD271 monoclonal antibody (Abcam Biochemicals, Cambridge, UK; ab31251) diluted 1:50 in 2% BSA and 2% goat serum (Sigma-Aldrich Ltd, Poole, UK) in PBS overnight at 4°C, washing three times in PBS/T and incubating with goat anti-mouse Alexafluor633 (Thermo Fisher Scientific, Paisley, UK) diluted 1:250 in 2% BSA and 2% goat serum in PBS containing diamidino-2-phenylindole dye (DAPI, Thermo Fisher Scientific, Paisley, UK) diluted 1:1000, for 1 hour at room temperature. Cover slips were mounted in hard set anti-fade mounting medium (Vector labs, Burlingame, California, USA) and analysed using a Leica TCS SP2 UV confocal microscope with LC2 2.61 software (Leica Microsystems, GmbH Heidelberg, Germany).

**Time-lapse confocal microscopy** – Melanoma cells expressing an LC3-RFP-GFP reporter construct were seeded into 6-well tissue culture plates at 1x10^5^ cells per well in 3 ml complete culture medium and allowed to attach overnight at 37^o^C. Medium was replaced with fresh complete culture medium containing DMSO, 16 nM trametinib, 5 μM chloroquine or the combination of trametinib and chloroquine, and time-lapse images taken every 15 minutes for 48 hours using a Nikon A1R confocal microscope (Nikon Instruments Europe BV, Amsterdam, The Netherlands) in an environmentally controlled hood at 37^o^C in a humidified atmosphere containing 5% CO_2_ in air.

**Clonogenic assay** – Melanoma cells were initially treated with 16 nM trametinib for 9 days prior to treatment for 48 hours with either DMSO, 16 nM trametinib, 10 μM chloroquine or the combination of trametinib and chloroquine. Cells were subsequently seeded in triplicate at 100, 250, 500 or 1000 cells per 10 cm tissue culture dish (Corning Inc., Corning, USA) in 7ml of fresh complete media and incubated at 37^o^C for 14 days. Emergent colonies were gently washed in PBS, fixed in 5 ml Carnoy’s fixative (100 ml acetic acid in 300 ml methanol) for 5 mins at room temperature, washed again in PBS and stained with 5 ml of 0.4% crystal violet (Sigma-Aldrich Ltd, Poole, UK) for 5 mins at room temperature. Colonies were counted using a colony counter (ColCount^TM^ Oxford Optronics, Abingdon, UK) and the cloning efficiency calculate as below:

| $\% cloning efficiency=\left( \frac{colonies counted}{cells seeded} \right)x100$ |  |
| --- | --- |

SiRNA-mediated knockdown of CD271 – Melanoma cells were seeded at 1.5x10^5^ cells per well in 6-well culture plates in 3ml of complete culture medium and allowed to attach overnight at 37^o^C. Three transfection mixtures were prepared and incubated for 30 mins at room temperature: 1) 2.5µl of lipofectamine RNAiMax reagent (Invitrogen, Paisley, UK) in 125µl Opti-MEM1x reduced serum medium (Life Technologies, New York, U.S.A) per well; 2) 2.5 µl of ON-TARGETplus Human NGFR siRNA (SMART pool siRNA, Dharmacon, GE lifesciences, [Lafayette, Colorado, U.S.A)](https://www.google.co.uk/search?biw=1920&bih=986&q=Lafayette+Colorado&stick=H4sIAAAAAAAAAOPgE-LSz9U3MM41SzI0UuIAsYssDS21tLKTrfTzi9IT8zKrEksy8_NQOFYZqYkphaWJRSWpRcUAAwbVmkQAAAA&sa=X&sqi=2&ved=0ahUKEwitqavpwLHMAhWIF8AKHV8FCnwQmxMIgAEoATAS) in 125 µl Opti-MEM1x reduced serum medium per target well; and 3) 2.5 µl Stealth RNAi™ siRNA Scrambled Control (Invitrogen, Paisley, UK) in 125 µl Opti-MEM1x reduced serum medium (Life technologies, U.S.A) per control well. Lipofectamine mixture was added to NGFR and Scrambled siRNA mixtures and incubated at room temperature for 15 minutes. Cells were washed in PBS prior to addition of 1 ml Opti-MEM1x reduced serum medium and 250 µl of respective lipofectamine/siRNA mixture and incubated at 37^o^C for 6 hours. The transfection was terminated by replacing the transfection mixture with 3 ml of fresh complete culture medium and knockdown efficiency determined after 24 or 72 hour incubation at 37^o^C.

**3D collagen-invasion assay** – Collagen Invasion assays were performed as previously described [13]. Briefly, A375 melanoma cells were treated for 42 days with 16 nM trametinib and transferred to 96-well tissue culture plates (Corning Incorporated, New York, USA) pre-coated with 100 µl per well 1.5% low melting point agarose (Sigma-Aldrich Ltd, Poole, UK) at 5000 cells per well in 200 µl complete culture medium and incubated for 72 hours at 37^o^C to form spheroids. A collagen mixture was prepared on ice by mixing 1008 µl Collagen I Bovine Protein (Thermo Fisher Scientific, Paisley, UK), 846 µl ddH_2_O, 224 µl Essential Modifed Eagle’s Medium (Lonza, Vervies, Belgium), 20 µl L-Glutamine (Sigma-Aldrich Ltd, Poole, UK), 68 µl 7.5% NaHCO_3_ (Sigma-Aldrich Ltd, Poole, UK) and 248 µl FCS (Sigma-Aldrich Ltd, Poole, UK). 200 µl of collagen mixture was added to 24-well tissue culture plates and allowed to solidify at 37^o^C for 5 mins prior to transfer of 3 melanoma spheroids per well in 300 µl of collagen mixture and incubation at 37^o^C for 5 mins to allow collagen to solidify around spheroids. 1 ml complete culture medium containing DMSO, 16 nM trametinib, 10 µM chloroquine, or trametinib and chloroquine was added to respective wells and images were captured using a Nikon A1R confocal microscope (Nikon Instruments Europe BV, Amsterdam, The Netherlands) after 0, 2, 4 and 7 days incubation at 37^o^C. Spheroid invasion was analysed using Volocity 3D Image Analysis Software (Volocity 6.3; PerkinElmer, Waltham, Massachusetts, USA) by measuring the average of 8 cross-sectional diameters for each image.

**Zebrafish xenograft assay** – Zebrafish eggs were collected from timed pair mating and incubated in 1X E3 media (50X E3 medium: 5.0 mM NaCl, 0.17 mM KCl, 0.33 mM CaCl_2_, 0.33 mM MgSO_4_ in 1 L ddH_2_O) at 28.5^o^C in air for 48 hours. Zebrafish embryos were then anaesthetized in 1.2 mM ethyl 3-aminobenzoate methanesulfonate (tricaine; Sigma-Aldrich Ltd, Poole, UK) in 1X E3 media and embedded in 1% low melting point agarose (Sigma-Aldrich Ltd, Poole, UK) in tricaine-containing 1X E3 media. A375 melanoma cells, previously treated for 42 days with 16 nM trametinib, were seeded at 2x10^6^ cells per T75 tissue culture flask in 10 ml complete culture medium containing 16 nM trametinib and incubated at 33^o^C for 24 hours to precondition cells for the xenograft assay. Medium was removed and cells were incubated for 20 minutes at 33^o^C in 5 ml of complete culture medium containing 25 µl 1,1'-Dioctadecyl-3,3,3',3'-tetramethylindocarbocyanine perchlorate (DiI, red fluorescent dye; Invitrogen, Paisley, UK), washed three times in PBS, trypsinised and resuspended in 20 µl PBS. Microinjection needles were made from borosilicate glass capillaries 3.3 (Hilgenberg, Malsfeld, Germany) using Model P-97 micropipette puller (Sutter instruments Co, Novato, USA) and attached to a microprocessor controlled Nanoliter 2000 injector system (World Precision Instruments, Hertfordshire, UK). Approximately 500 Dil-labelled melanoma cells in a volume of 5 nl were injected into the inferior section of the yolk sac of anaesthetized, embedded zebrafish embryos. Fish were examined for the presence of melanoma cells within the yolk sac using a fluorescent stereo Leica M165 FC microscope (Leica Microsystems, GmbH Heidelberg, Germany). Fish with a single localised group of melanoma cells were carefully removed from the agarose/tricaine solution. Selected fish were randomly chosen, full-depth images of each fish at 0 hour were captured using an inverted Leica SP8 confocal microscope (Leica Microsystems, GmbH Heidelberg, Germany) at 488 nm (green blood vessels) and 564 nm (red cells), and fish were placed individually into a 24-well plate in 1 ml of E3 media containing either DMSO, 16 nM trametinib, 5 µM PIK-III or the combination of trametinib and PIK-III. Fish were incubated at 33^o^C in a humidified atmosphere of air for 72 hours and repeat images of each fish were taken as previously described. Images were analysed using Volocity 3D Image Analysis Software (Volocity 6.3; PerkinElmer, Waltham, Massachusetts, USA) to measure cell movement. Extended focus z-stack confocal images of zebrafish embryos were used to manually measure the two-dimensional distance moved by individual DiI-positive melanoma cells from site of injection.

Statistical Analysis – Data for mean +/- 95% confidence intervals (CI) were analysed by two-sided tests using Statistical Prism 7 software (Graph Pad, San Diego, CA USA) and p-values of *^/#^P< 0.05, **^/##^P< 0.01, ***P< 0.001, ****P<0.0001 were regarded as statistically significant. Exact p-values are reported where p is greater than 0.001. Western blot protein expression data was acquired and analysed using Odyssey FC Image studio software (LI-COR Biotechnology UK Ltd, Cambridge, UK), expression intensity of target bands were normalized to the corresponding loading control (β-actin or GAPDH) and presented as the mean of three individual replicate experiments relative to a control condition. Data deriving from all MTS, MTT and ATP release assays were reported as the mean of a minimum of four replicates for 3 separate experiments and compared using a one-way ANOVA with Tukey’s multiple comparison tests. Data from MTS cell viability assays of isolated CD271-positive and -negative subpopulations were analysed using a Mann Whitney U non-parametric T-test. Immunohistochemical expression of CD271 and p62 for each FFPE sample is reported as the mean percentage staining intensity of 10 representative areas and compared using one-way ANOVA with Tukey’s multiple comparison tests. Invasion of all visible DiI-positive melanoma cells within zebrafish embryos were measured for at least 2 fish per treatment condition, for 5 independent experiments, and conditions were compared using Kruskal-Wallis non-parametric rank coefficient with Dunn’s multiple comparison tests.
