# Supplementary figures and images for: Harnessing autophagy to overcome mitogen‐activated protein kinase kinase inhibitor‐induced resistance in metastatic melanoma
Source: Br J Dermatol. 2018 Nov 25;180(2):346–56. doi: 10.1111/bjd.17333 (PMC7816093; doi:10.1111/bjd.17333)

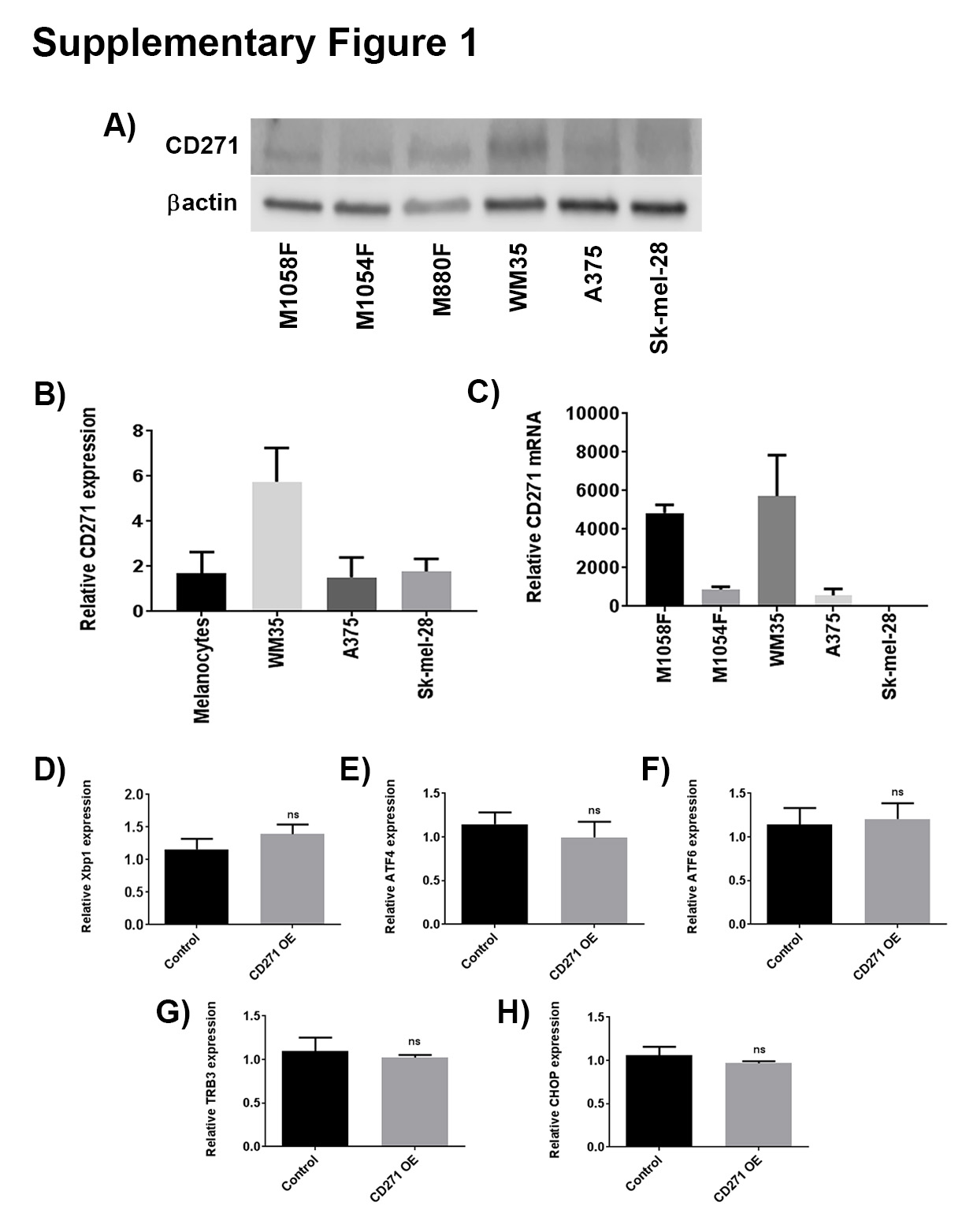

Supplement: Supplementary file 2 — Fig S1. (a) Representative Western blot analysis of CD271 and β‐actin expression in primary human melanocytes (M1058F, M1054F, M880F) and human metastatic melanoma cell lines (WM35, A375 and SKmel28). (b) Densitometry quantification of CD271 expression and (c) quantification of CD271 mRNA transcript levels in primary human melanocytes and human metastatic melanoma cell lines (n = 3 independent Western blot replicates). (d–h) Relative expression of Xbp1, ATF4, ATF6, TRB3 and CHOP mRNA transcript level in A375 melanoma cells flowing over expression of CD271 (CD271 OE). [file BJD-180-346-s002.jpg]

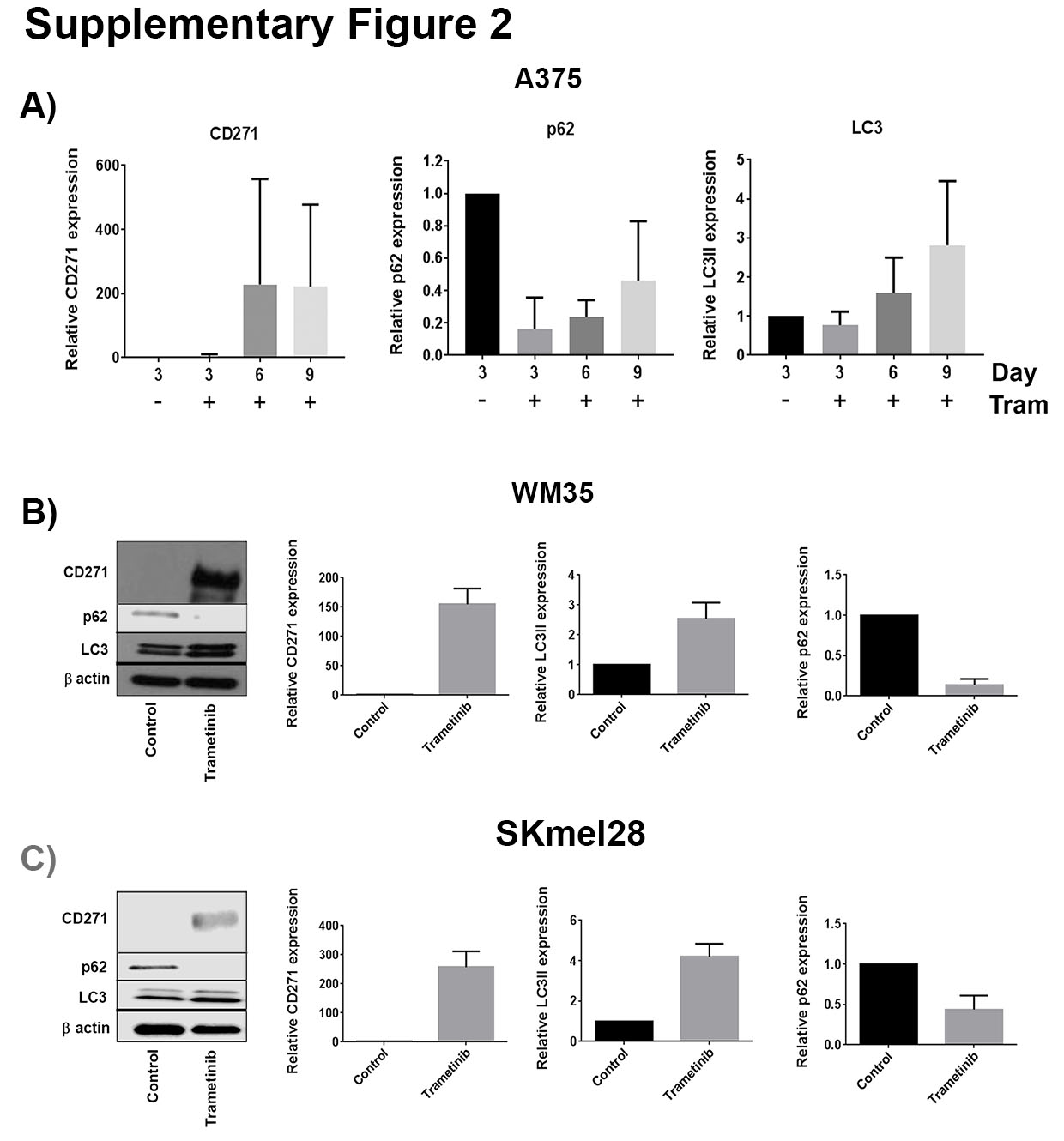

Supplement: Supplementary file 3 — Fig S2. (a) Densitometry quantification of CD271, p62 and LC3‐II expression by A375 cells following treatment with dimethyl sulfoxide or 16 nmol L−1 trametinib for 3, 6 or 9 days. (b, c) Representative Western blot analysis of CD271, p62 and LC3, and densitometry quantification of CD271, LC3‐II and p62 expression (n = 3 independent Western blot replicates), by WM35 and SKmel28 cells. [file BJD-180-346-s003.jpg]

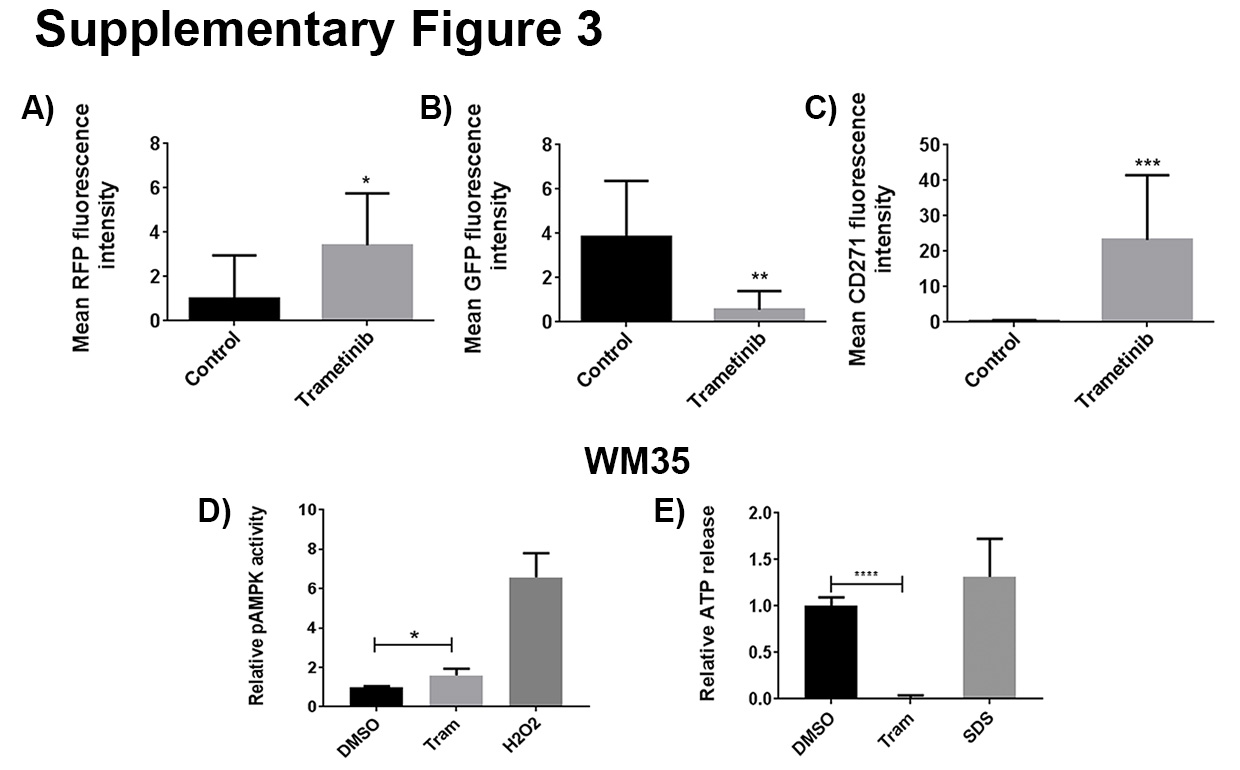

Supplement: Supplementary file 4 — Fig S3. (a–c) Mean fluorescence intensity of LC3–red fluorescent protein (RFP), LC3–green fluorescent protein (GFP) and CD271–A647 staining by WM266‐4 cells following treatment with 16 nmol L−1 trametinib for 9 days [n = 10 images; mean ± 95% confidence interval (CI), Mann–Whitney test (*P = 0·012, **P = 0·002, ***P < 0·001)]. (d) Relative phospho‐adenosine monophosphate‐activated protein kinase (AMPK) activity or (e) adenosine triphosphate (ATP) release by WM35 cells following treatment for 9 days in the presence or absence of 16 nmol L−1 trametinib, or 10 min in the presence of either 1 mmol L−1 H2O2 or 10% sodium dodecyl sulfate (SDS) as positive controls [n = 3 independent experiments; mean ± 95% CI, one‐way anova with Tukey's post‐hoc correction, (*P = 0·042, ****P < 0·001)]. [file BJD-180-346-s004.jpg]

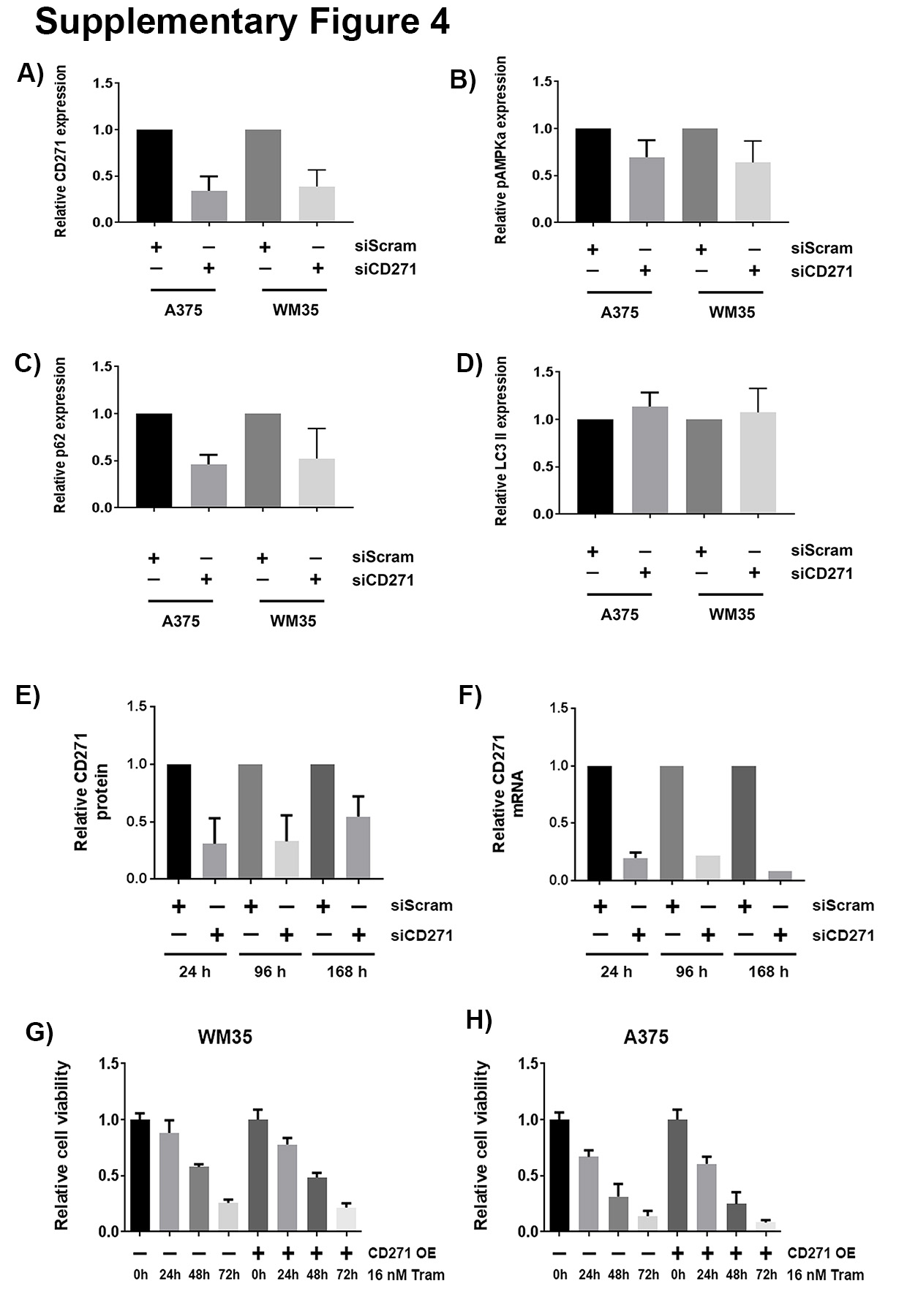

Supplement: Supplementary file 5 — Fig S4. (a–d) Densitometry quantification of CD271, phospho‐adenosine monophosphate‐activated protein kinase (AMPK), p62 and LC3‐II expression by A375 and WM35 cells following treatment with 16 nmol L−1 trametinib for 9 days and then for a further 72 h following transfection with scrambled nontarget (siScram) or CD271 (siCD271) small interfering RNA (siRNA; n = 3 independent Western blot replicates). (e) Densitometry quantification of CD271 and (f) quantification of CD271 mRNA transcript levels in WM35 cells after 24, 96 and 168 h after transfection with siScram or siCD271. (g, h) Relative viability, as measured by MTS metabolism, of WM35 and A375 wild‐type cells or cells overexpressing CD271, following treatment in the presence or absence of 16 nmol L−1 trametinib for 24, 48 or 72 h. [file BJD-180-346-s005.jpg]

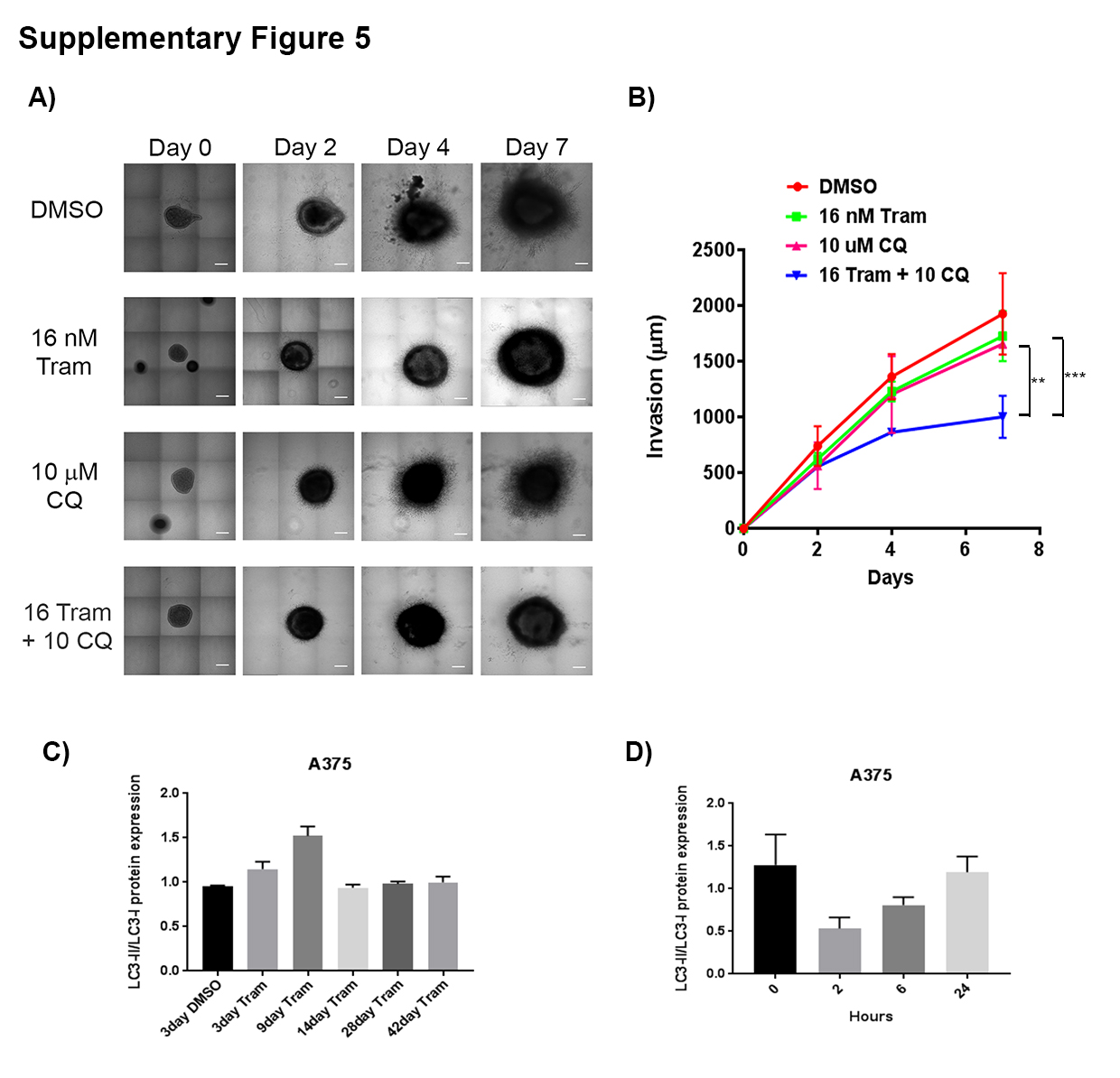

Supplement: Supplementary file 6 — Fig S5. (a) Representative photomicrographs of A375 spheroids in type I collagen following treatment with 16 nmol L−1 trametinib for 42 days, and subsequently treated with dimethyl sulfoxide (DMSO), 16 nmol L−1 trametinib, 10 mmol L−1 chloroquine or the combination of 16 nmol L−1 trametinib and 10 mmol L−1 chloroquine [16 Tram + 10 chloroquine (CQ)] for 0, 2, 4 or 7 days; ×10 magnification, scale bar = 100 μm. (b) Invasion of A375 trametinib‐resistant spheroids relative to size at day 0 following the same treatment protocol as for (a) [n = 24 cross‐sectional diameters at each time point from three independent experiments; mean ± SD, two‐way anova with Tukey's post‐hoc correction (**P = 0·002, ***P < 0·001)]. (c) Densitometric quantification of the ratio of LC3‐II/LC3‐I expression by A375 cells treated for 3 days with DMSO, or for 3, 9, 14, 28 and 42 days with 16 nmol L−1 trametinib (n = 3 independent Western blot replicates). (d) Densitometric quantification of the ratio of LC3‐II/LC3‐I expression by A375 cells treated for 0, 2, 6 or 24 h with 5 mmol L−1 PIK‐III (n = 3 independent Western blot replicates). [file BJD-180-346-s006.jpg]
